# Supplementary material for: Safety and Perioperative Outcomes of Laparoscopic vs. Open Hepatectomy of Central-Located Liver Lesions: A Multicenter, Propensity Score-Matched, Retrospective Cohort Study
Source: J Clin Med. 2023 Mar 10;12(6):2164. doi: 10.3390/jcm12062164 (PMC10057074; doi:10.3390/jcm12062164)
Supplement: Supplementary file 1 [file jcm-12-02164-s001.zip › jcm-2191445-supplementary.pdf]

Safety and perioperative outcomes of laparoscopic vs. open hepatectomy of central-located liver lesions: A multicenter, propensity score-matched, retrospective cohort study

Bao Jin1#, Muyi Yang2#, Yinhan Wang1,3#, Gang Xu1,4, Yuxin Wang1, Yuke Zhang1,3, Yitong Liu1,3, Xinming Niu1,3, Xiao Liu1, Xueshuai Wan1, Huayu Yang1, Xin Lu1, Xinting Sang1, Yilei Mao1, Zhixian Hong2*, Shunda Du1*

[1] Department of Liver Surgery, Peking Union Medical College (PUMC) Hospital, PUMC & Chinese Academy of Medical Sciences, Dongcheng, Beijing, 100730, China.

[2] Department of Hepatobiliary Surgery, Fifth Medical Center of Chinese PLA General Hospital, Fengtai, Beijing, 100039, China.

[3] Peking Union Medical College (PUMC), Chinese Academy of Medical Sciences & PUMC, Dongcheng, Beijing, 100730, China.

[4] Department of Liver Surgery and Liver Transplantation Centre, West China Hospital, Sichuan University, Chengdu, Sichuan Province, 610041, China.

[#] These authors contributed equally to the article.

* Correspondence:

Zhixian Hong Email: zqyhzx@sina.com

Shunda Du E-mail: dushd@pumch.cn

Supplementary Table 1 Reported studies of laparoscopic mesohepatectomy

No.	First author	Year	Country	Type	Sample size	Type of lesion	Tumor size (cm)	Operation time (min)	EBL (ml)	POS (days)	Morbidity/ mortality rate	Ref
1	M A Machado	2011	Brazil	Case report	1	colorectal liver metastasis	NR	200 min	minimum	5	0	[14]
2	Claudius Conrad	2015	France	Retrospective cohort study	32	31 with malignancies,1 with liver adenoma	NR	253 min (SD 94)	403 (SD 475)	10.4 days	44%/0	[12]
3	Wan-Joon Kim	2017	Korea	Case series	10	8 with HCC, 2 with colorectal liver metastasis	2.9±1.9	330±92.7	325±234.5	9.5±3.4	10%/0	[22]
4*	Wei Li	2019	China	Retrospective	96/32	HCC	5.7 ± 3.0	175.0 ± 37.3	395.8 ±	12.8 ±	0.3%/0,	[13]

				cohort control study			/4.0 ± 2.0	/173.3 ± 55.1	361.9/ 328.2 ± 328.0	5.1/10.8 ± 4.1	21.5%/14.6%	
5	S W Zhu	2019	China	Case series	40	Malignancies	6.2±2.9	333±30	173±129	10.7±2.7	7.5%/0	[15]
6	Giammauro Berardi	2019	Japan	Case report	1	HCC	3	420	261	8	0/0	[21]
7	Carolijn Antoine Camerlo	2021	France	Case report	1	HCC	7	NR	NR	8	0/0	[10]
8	Rui Chen	2021	China	Case report	1	HCC	13	360	200	8	0/0	[11]

EBL, estimated blood loss; POS，post-operative hospital stay.

*The two sets of data in the fourth study (Wei Li, 2019) represent the open group (front) and the laparoscopy group (rear), respectively.
